# Validation and comparison of the Arabic versions of GOHAI and OHIP-14 in patients with and without denture experience

**DOI:** 10.1186/s12903-018-0620-5

**Published:** 2018-09-17

**Authors:** Sarah M. Osman, Nadia Khalifa, Mohammed Nasser Alhajj

**Affiliations:** 1 Department of Oral Rehabilitation, Faculty of Dentistry, University of Khartoum, Khartoum, Sudan; 2 Department of Preventive and Restorative Dentistry, Faculty of Dental Medicine, University of Sharjah, Sharjah, United Arab Emirates; 3 Department of Prosthodontics, Faculty of Dentistry, Thamar University, Dhamar, Yemen

**Keywords:** Complete denture, GOHAI, OHIP-14, Oral health-related quality of life, Sudanese

## Abstract

**Background:**

The assessment of oral heath related quality of life (OHRQoL) for complete denture wearers according to a participant’s subjective perception may provide an indication of the adaptive capacity of the individual. The aim of this study was to compare and assess the validation of two quality of life measures, the Oral Health Impact Profile-14 (OHIP-14) and Geriatric Oral Health Assessment Index (GOHAI), in patients with and without previous denture experience.

**Methods:**

A total of 69 elders (36 male and 34 female; mean age, 63 years) from Dental Clinics of the University of Khartoum and the National University in Sudan, with and without previous denture experience, were included in the study. OHRQoL was assessed using two Arabic-translated questionnaires (OHIP-14 and GOHAI) before and after complete denture therapy. Global self-ratings of oral and general health were obtained, and denture satisfaction was assessed using the Denture Satisfaction questionnaire.

**Results:**

Both tools had significant correlations with self-rating oral health in patients without denture experience (*P* < 0.05). However, no significant correlations were found in patients with denture experience. There were significant differences between pre-and post-treatment total scores with both the GOHAI and OHIP-14 (*P* < 0.001). Responsiveness to treatment using GOHAI and OHIP-14 revealed overall mean effect size higher in patients without (1.49) and (0.83) than those with previous denture experience (0.89) and (0.60), respectively. However, neither tool could detect significant differences between patients with and without denture experience (*P* > 0.05).

**Conclusion:**

Greater improvements of OHRQoL after complete denture therapy were observed in participants without than those with previous denture experience. The Arabic-translated versions of GOHAI and OHIP-14 can be regarded as effective measures for assessing treatment outcomes of complete denture therapy.

## Background

Globally, attention has been focused towards self-perception of an individual’s oral health, and the effect of oral diseases on their functional and psychological well-being. A range of oral heath related quality of life (OHRQoL) instruments have been used to assess the functional and psychosocial impacts of oral diseases [1]. Tooth loss can have a deleterious effect on oral function and affect quality of life [2, 3]. It can be assumed that provision of prosthodontic treatment, such as complete dentures, has a positive impact on oral health. The ability of the tool to outline the impact of an intervention is a key measurement property of any OHRQoL measure [4]. The Oral Health Impact Profile-14 (OHIP-14) and the Geriatric/General Oral Health Assessment Index (GOHAI) questionnaires are popular tools for assessing self-perception of OHRQoL. The OHIP-14 questionnaire was developed and validated by Slade and Spencer [5]. The GOHAI was initially proposed in English by Atchison and Dolan for the US population, and used to measure the oral health problems of elders [6]. Both the OHIP-14 and GOHAI questionnaires have been proposed for utilisation in clinical trials and epidemiological surveys, evaluation of treatment outcome for population health measurements, and provision of information for policy decisions [6, 7].

Currently, it is recognised that if measures are to be used across cultures, the items must be translated well linguistically and adapted culturally to maintain the content validity of the instrument at a conceptual level across different cultures [8]. Both questionnaires have been translated into different languages, validated, and culturally adapted for different countries [9–13]. Edentulous subjects are in need of teeth replacements to increase their ability to masticate food, speak properly, and to improve aesthetics and psychological well-being. The evaluation of OHRQoL using the GOHAI and OHIP-14 before and after treatment may demonstrate the need of elders requiring tooth replacement with dentures and provide indications of the quality of the serviced treatment. This in turn will benefit the dental profession as well as policy makers. The study based on the hypothesis that participants with denture experience will response better than those without denture experience. Therefore, this study investigated the responsiveness of complete denture therapy in patients with and without previous denture experience using the OHIP14 and GOHAI as outcome measures.

## Methods

This study included participants aged ≥40 years seeking treatment at the Prosthodontic Department Clinics of the University of Khartoum and the National University in Khartoum, Sudan. Those requiring conventional complete dentures for both jaws were included in the study. Conventional complete dentures were made by undergraduate students under the supervision of clinical instructors. Participants with cognitive impairment, motility disorders, serious illness, temporomandibular disorder, or mental retardation were excluded from study. Those from the suburbs were also excluded due to recalling difficulties. Approval from the Ethical Committee of the University of Khartoum (Faculty of Dentistry) was obtained prior to the study. Written informed consent was obtained from all the participating patients.

Applying the formula $n=\frac{N}{1+{Ne}^2}$ to the target population of 65, including only those who satisfied the criteria, yielded a sample size of 56 participants. The target population of 65 was based on 3 months monitoring of subjects seeking for treatment with conventional complete dentures for both jaws. The sample size was increased by 20% to reduce the impact of loss to follow-up on study power, resulting in a sample of 69 patients. Data were collected using a structured face-to-face interview administered by the first researcher in order to collect sociodemographic information (including sex, age, marital status, and educational level), self-rating of oral and general health, presence or absence of systemic disease, use of long-term use medications, previous denture experience, participants’ satisfaction with dentures, and OHRQoL using the OHIP-14 and GOHAI. The OHIP-14 questionnaire used in this study was previously translated into a the Arabic version, and was found to have good validity and reliability [13]. It is a 14-item questionnaire in which participants are asked to respond according to the frequency of impacts on a 5-point Likert scale (never, seldom, sometimes, fairly often, and very often). The GOHAI questionnaire, however, comprises only 12 items, and similar to the OHIP-14, answers are recorded on a 5-point Likert scale.

### GOHAI questionnaire

The GOHAI was translated from English to Arabic, using Sudanese colloquial language. The translation was performed following guidelines described in previous studies [12, 13]. A forward and backward translation approach was adopted because it was used successfully for the translation of the OHRQoL measures. Forward translation was performed by establishing two teams of bilingual dentists, each team comprising two translators in order to produce two sets of Arabic copies from the principle questionnaire. A revised Sudanese-Arabic copy was then produced by both teams. Working from the revised Sudanese-Arabic version of the questionnaire, two bilingual dentists performed the backward translation without access to the English version. Two Arabic linguistic experts and the forward translators then re-evaluated all the translations to achieve a concurrence on aspects of divergence.

### Denture satisfaction (DS) questionnaire

This questionnaire comprised 12 items and was administered to patients by face-to-face interview 1 month after complete denture insertion by the same calibrated dentist. The subjects responded “satisfied”, “regular”, or “dissatisfied” for each item. They evaluated satisfaction with retention, comfort, stability, ability to speak, and overall satisfaction with maxillary and mandibular complete dentures. Data were encoded, and scores of 2, 1, or 0 were recorded. This questionnaire aimed to evaluate patient’s satisfaction with complete denture treatment and exclude the probability of lowering OHRQoL perception by having low quality prosthesis. A forward-backward approach was used to create an Arabic version of the Denture Satisfaction Questionnaire, and translation guidelines were followed as prescribed for GOHAI questionnaire translation.

A sample of ten participants were recruited, and the GOHAI, OHIP-14 and Denture Satisfaction questionnaires were piloted. All questionnaires were then administered twice with a 2 weeks interval between the administrations in order to assure validity and reliability.

OHIP-14 and GOHAI questionnaires were administered for the first time at the denture insertion appointment preceding the insertion of the denture, and the second time it was performed after 1 month. At the same time (1 month after insertion), the denture satisfaction (DS) questionnaire was administered to the participants.

### Data analysis

Data were analysed with descriptive statistics in terms of frequency distributions and charts according to denture experience (Group A: patients with denture experience and Group B: patients without denture experience). Statistical significance was accepted when *p* < 0.05. Data analysis was performed using IBM SPSS Statistics for Windows version 22.0 (IBM Corp., USA). Mean GOHAI and OHIP-14 scores were assessed for demographic variables using the Mann Whitney and Kruskal Wallis tests as appropriate.

For statistical purposes, the total scores of the instruments were calculated by summing the items with responses. Therefore, the total scores ranged from 0 to 12 for GOHAI and DS and from 0 to 14 for OHIP-14. Moreover, the additive (ADD) count method was used to calculate GOHAI and OHIP-14 scores. ADD scores for the GOHAI were obtained by summing the response codes for the 12 items after reversing the coding of the three positively worded items (swallowing, appearance, and discomfort when eating) [14]. For the OHIP-14, they were obtained by summing the response codes to the 14 items constituting the measure. Responses were scored on a scale ranging from 0 to 4. Therefore, the summary scores ranged from 0 to 48 for the ADD-GOHAI and from 0 to 56 for the ADD-OHIP-14, with a lower score indicating better oral health [15].

#### Reliability

Internal consistency was explored using Cronbach’s alpha and the reliability was assessed using intraclass correlation coefficient (ICC) test for correlation between readings and paired t-test for differences between readings with scores from repeated administrations of the GOHAI to ten patients 2 weeks after the first administration. Any interval between administrations of questionnaires can be considered a compromise.

#### Validity

Validity test was performed to ensure that the instrument was quantifying what it was intended to measure. Data from the first administration was used to assess the validity of the GOHAI and OHIP-14. Two types of construct validity tests were performed: convergent and discriminant validity. Convergent validity describes how closely a measure is related to other measures of the same construct, and was evaluated by identifying associations between perceived oral and general health status and GOHAI and OHIP-14 scores using Spearman’s correlation coefficient. For convergent validity, we hypothesised that lower GOHAI and OHIP-14 scores would be associated with good self-reported oral and general health. Self-perceived oral health assessment was performed by asking the subjects “How do you rate your oral health?” answers were provided on a 3-point ordinary Likert scale (“good”, “fair”, or “poor”). The same responses were provided with self-perceived general health question “How do you rate your general health?” Another test of validity was performed from data of the second administration between GOHAI and OHIP-14 total scores and DS total scores.

Discriminant validity assesses the extent to which a scale can distinguish between groups with known differences. GOHAI and OHIP-14 total scores and sub-scale domains were compared according to denture experience. For discriminant validity, we hypothesised that lower GOHAI and OHIP-14 scores would be associated with denture experience. Comparison between the study variables according to denture experience was also performed.

#### Responsiveness

The responsiveness that emerged from comparing pre- and post-treatment scores was assessed for both instruments. The association with denture experience and GOHAI/OHIP-14 score was examined using a paired sample t-test. The total scores and sub-scale domains of both instruments were used for responsiveness test and the effect size was also calculated.

## Results

At baseline (first administration), the sample comprised 69 participants (group A = 36 and group B = 33) requesting complete dentures at the Prosthodontic Clinics of the Faculty of Dentistry University of Khartoum, and the National University. After a 1-month interval from baseline, 60 participants (group A = 33 and group B = 27) completed the questionnaires at the second administration. The mean age of the participants was 65.47 ± 7.13 years while it was 61.67 ± 8.00 years for participants from group B. The majority of participants were in the 60–69 age group, with the least were in the ≥80 years among both groups. Out of the 36 participants in group A, 21 were males and 15 were females while, females were more than males in group B (54.5 and 45.5%, respectively). Regarding participant marital status, most of the participants in both groups (more than 70%) were married. Information collected on their educational level revealed that majority of participants from both groups just completed primary school education. Regarding medical status, 23 (63.9%) subjects from group A had no systemic diseases and 22 (61.1%) subjects were taking medications. For group B, 57.6% (19 subjects) presented with systemic diseases and 45.5% (15 subjects) were taking medications. Approximately half the study population (52.2%) had previous denture experience ranging from 6 months to 10 years, with one participant having 30 years of denture experience (Table 1).

**Table 1:** Sample characteristics according to denture experience

Variable		Denture experience	
		Yes	No
Age group	50–59	5(13.9)	12 (36.4)
	60–69	19 (52.8)	15 (45.5)
	70–79	10 (27.8)	5 (15.2)
	80–89	2 (5.6)	1 (3.0)
Gender	Male	21 (58.3)	15 (45.5)
	Female	15 (41.7)	18 (54.5)
Marital status	Single	2 (5.6)	1 (3.0)
	Married	28 (77.8)	24 (72.7)
	Divorced	2 (5.6)	2 (6.1)
	Widowed	4 (11.1)	6 (18.2)
Educational level	Illiterate	5(13.9)	5 (15.2)
	khalwa	1 (2.8)	3 (9.1)
	Primary school	16 (44.4)	16 (48.5)
	senior high school	7 (19.4)	7 (21.2)
	Graduate	7 (19.4)	2 (6.1)
Perceived general health	Good	18 (50.0)	20 (60.6)
	Moderate	15 (41.7)	12 (36.4)
	Poor	3 (8.3)	1 (3.0)
Perceived oral health	Good	22 (61.1)	20 (60.6)
	Moderate	11 (30.6)	8 (24.2)
	Poor	3 (8.3)	5 (15.2)
Systemic disease	Yes	13 (36.1)	19 (57.6)
	No	23 (63.9)	14 (42.4)
Medication long use	Yes	14 (38.9)	15 (45.5)
	No	22 (61.1)	18 (54.5)
Patient’s age by years	Mean ± SD	65.47 ± 7.13	61.67 ± 8.00
GOHAI total score	Mean ± SD	14.00 ± 8.76	16.55 ± 8.54
OHIP total score	Mean ± SD	7.17 ± 9.32	11.30 ± 11.58
DS total score	Mean ± SD	21.18 ± 3.50	20.63 ± 4.90

No significant difference (*P* = 0.962) was found between group A and B regarding denture satisfaction. The overall satisfaction with maxillary and mandibular dentures was reported as 91.6 and 61.65%, respectively. Almost all patients were satisfied with the retention, stability, comfort, and appearance of their maxillary dentures. Dissatisfaction responses were mainly related to mandibular dentures. Self-perception of oral and general health was measured using a single-item self-rating response. For both groups, most participants rated their oral health as good, 8.3% from group A and 15.2% from group B had poor oral health. Similarly, self-perception of general health was reported as good from most participants of both groups, 8.3% from group A and only one participant (3.0%) from group B reported their general health as poor (Table 1).

### Reliability

The mean intraclass correlation coefficient for reliability among the 10 participants who underwent assessment of the GOHAI on two occasions was 0.893 ± 0.083. According to Shrout and Fleiss [16], an intraclass correlation coefficient > 0.75 indicates excellent reliability. A paired sample t-test was also used to examine test-retest reliability. The mean difference was − 0.074, and there was no significant difference between GOHAI-1 and GOHAI-2 (*P* > 0.05). For internal consistency, the Cronbach alpha of the GOHAI was 0.711 for group A and 0.713 for group B. For both groups, the Cronbach α for the GOHAI was 0.710, and α values for the seven sub-scales ranged from 0.638 to 0.718 if items were deleted one by one. According to Bland and Altman, a Cronbach α of 0.70–0.80 is desirable [17].

### Validity

#### Convergent validity

Spearman correlation was used to examine the relationship between ratings of oral and general health and GOHAI and OHIP-14 total scores from the first administration (*n* = 69) for both groups. In addition, the GOHAI and OHIP-14 total scores from the second administration (*n* = 60) for both groups were tested for correlation with DS total score. For group A, GOHAI total score had no significant correlation with self-rating oral and general health neither with DS total score (*P* > 0.05). However, OHIP-14 total score had significant correlation with DS total score (*r* = − 0.448; *P* = 0.009) and self-rating general health (*r* = 0.348; *P* = 0.037) but, similar to GOHAI, no significant correlation was found with self-rating oral health (*P* > 0.05). For group B, significant correlation was found between both GOHAI and OHIP-14 with DS total score. Similarly, both instruments were significantly associated with self-rating oral health. However, no significant correlations were found between both instruments and self-rating general health (Table 2).

**Table 2:** Correlation between GOHAI and OHIP with perceived general and oral health and denture satisfaction

	Denture experience							
	Yes				No			
	Perceived general health	Perceived oral health	GOHAI total score	OHIP total score	Perceived general health	Perceived oral health	GOHAI total score	OHIP total score
Perceived general health	1.000				1.000			
Perceived oral health	.184	1.000			.218	1.000		
	.282				.223			
GOHAI total score	.141	.189	1.000		.108	.356^a^	1.000	
	.412	.269			.551	.042		
OHIP total score	.348^a^	.183	.723^b^	1.000	.252	.356^a^	.752^b^	1.000
	.037	.286	.000		.158	.042	.000	
DS total score			−.168	−.448^b^			−.739^b^	−.737^b^
			.350	.009			.000	.000

^a^Correlation is significant at the 0.05 level (2-tailed); ^b^Correlation is significant at the 0.01 level (2-tailed)

#### Discriminant validity

Surprisingly and in contrast to what we had expected, there was no significant differences between total scores of both instruments (GOHAI and OHIP) when used to distinguish the difference between subjects with and without denture experience. Apart from social disability domain of the OHIP-14 which had significant difference (*P* = 0.011) between both groups, all other domains of both instruments had no significant differences (Tables 3 and 4). As shown in Table 5, the results of comparison between the study variables according to denture experience revealed no significant differences between the study groups except for systemic disease and long use of medication. For the former, OHIP-14 could detect a significant difference (*P* = 0.037) in patients with denture experience (group A) while GOHAI could detect a significant difference (*P* = 0.033) in patients without denture experience (group B). For the latter, both GOHAI and OHIP-14 could detect significant differences in group B subjects (*P* = 0.011 and *P* = 0.029, respectively).

**Table 3:** comparison between GOHAI and OHIP-14 by domains and total scores before treatment

		Denture experience (*n* = 69)		P
		Yes	No	
GOHAI	Physical function	7.22 ± 4.56	9.12 ± 4.59	.084
	Pain and discomfort	2.86 ± 2.61	2.42 ± 3.09	.362
	Psychosocial impacts	3.92 ± 4.40	5.00 ± 4.96	.387
	Total score	14.00 ± 8.76	16.55 ± 8.54	.229
OHIP-14	Functional limitation	1.19 ± 1.91	1.42 ± 1.89	.520
	Physical disability	1.67 ± 2.16	1.82 ± 2.16	.651
	Physical pain	1.86 ± 2.63	2.61 ± 3.30	.500
	Psychological discomfort	0.81 ± 1.64	1.36 ± 1.93	.149
	Psychological disability	0.83 ± 1.65	1.91 ± 2.57	.059
	Social disability	0.33 ± 1.39	1.09 ± 1.89	.011^*^
	Handicap	0.47 ± 1.56	1.09 ± 2.07	.089
	Total score	7.17 ± 9.32	11.30 ± 11.58	.149

*Significance at the 0.05 level (2-tailed)

**Table 4:** comparison between GOHAI and OHIP-14 by domains and total scores after treatment

		Denture Experience (*n* = 60)		P
		Yes	No	
GOHAI	Physical function	3.94 ± 4.80	2.81 ± 3.90	.449
	Pain and discomfort	1.52 ± 2.00	1.15 ± 1.79	.453
	Psychosocial impacts	1.15 ± 2.22	1.04 ± 2.12	.985
	Total score	6.61 ± 6.95	5.00 ± 5.92	.502
OHIP-14	Functional limitation	0.30 ± 0.85	0.37 ± 1.01	.763
	Physical disability	0.70 ± 1.19	0.52 ± 0.98	.633
	Physical pain	0.55 ± 1.18	0.33 ± 0.83	.501
	Psychological discomfort	0.21 ± 0.86	0.67 ± 1.39	.078
	Psychological disability	0.21 ± 0.86	0.22 ± 0.70	.535
	Social disability	0.00 ± 0.00	0.22 ± 0.70	.051
	Handicap	0.06 ± 0.35	0.04 ± 0.19	.905
	Total score	2.03 ± 2.76	2.37 ± 3.43	.752

**Table 5:** Comparison between the study variables according to denture experience

Variable		Denture experience			
		Yes		No	
		GOHAI	OHIP	GOHAI	OHIP
Age group	50–59	12.40 ± 2.70	4.60 ± 4.93	18.17 ± 8.75	14.17 ± 13.62
	60–69	14.79 ± 8.66	6.16 ± 6.37	16.33 ± 9.36	12.07 ± 10.99
	70–79	13.30 ± 11.77	10.60 ± 14.80	15.00 ± 6.04	4.40 ± 4.62
	80–89	14.00 ± 5.66	6.00 ± 8.49	8.00 ± 00	0.00 ± 00
	P-value	.824	.830	.670	.197
Gender	Male	13.33 ± 8.75	6.86 ± 11.17	14.47 ± 7.60	7.53 ± 8.76
	Female	14.93 ± 8.99	7.60 ± 6.23	18.28 ± 9.09	14.44 ± 12.89
	P-value	.809	.249	.232	.088
Marital status	Single	25.50 ± 21.92	25.00 ± 35.36	5.00 ± 00	7.00 ± 00
	Married	13.07 ± 6.84	6.00 ± 5.86	16.71 ± 8.00	10.92 ± 11.58
	Divorced	14.50 ± .71	8.00 ± 5.66	11.50 ± 7.78	3.50 ± 4.95
	Widowed	14.50 ± 14.75	6.00 ± 7.12	19.50 ± 10.62	16.17 ± 13.38
	P-value	.841	.892	.272	.571
Educational level	Illiterate	12.60 ± 2.19	6.00 ± 8.25	14.20 ± 9.76	10.00 ± 6.44
	khalwa	0.00 ± 00	4.00 ± 00	19.67 ± 2.08	10.67 ± 8.50
	Primary school	14.94 ± 9.41	7.56 ± 5.90	17.00 ± 9.47	13.63 ± 14.40
	senior high school	11.29 ± 6.29	3.29 ± 5.25	15.00 ± 9.13	8.71 ± 10.58
	Graduate	17.57 ± 11.12	11.43 ± 17.47	19.50 ± 2.12	6.00 ± 0.00
	P-value	.485	.490	.813	.874
Perceived general health	Good	14.00 ± 10.68	6.28 ± 11.94	16.10 ± 9.26	9.35 ± 10.79
	Moderate	12.93 ± 5.80	7.73 ± 5.68	16.83 ± 7.80	12.25 ± 10.56
	Poor	19.33 ± 9.29	9.67 ± 8.14	22.00 ± 00	39.00 ± 00
	P-value	.486	.118	.732	.161
Perceived oral health	Good	12.23 ± 8.01	5.41 ± 5.85	14.10 ± 7.29	7.35 ± 8.82
	Moderate	17.00 ± 10.19	10.91 ± 14.32	18.88 ± 10.62	16.00 ± 14.20
	Poor	16.00 ± 7.81	6.33 ± 4.93	22.60 ± 6.84	19.60 ± 11.93
	P-value	.499	.520	.114	.131
Systemic disease	Yes	15.15 ± 8.96	9.15 ± 6.57	13.58 ± 5.76	7.74 ± 8.80
	No	13.35 ± 8.78	6.04 ± 10.54	20.57 ± 10.16	16.14 ± 13.38
	P-value	.541	.037^*^	.033^*^	.111
Medication long use	Yes	12.93 ± 6.62	7.57 ± 6.55	12.27 ± 4.93	5.53 ± 4.81
	No	14.68 ± 9.98	6.91 ± 10.87	20.11 ± 9.36	16.11 ± 13.40
	P-value	.637	.324	.011^*^	.029^*^

*Significance at the 0.05 level (2-tailed)

#### Responsiveness

Denture experience tended to be positively related to both GOHAI and OHIP-14 scores. There was a greater improvement in the OHRQoL of patients without previous denture experience (mean difference = 12.96 ± 10.90) than those with previous denture experience (mean difference = 7.90 ± 9.43). In both groups, the difference was significant but, this significance was higher with GOHAI (*P* < 0.001 for both groups) than with OHIP-14 (*P* = 0.002 for group A; *P* = 0.001 for group B). Using the GOHAI, the responsiveness to treatment, shown by the overall effect size, was higher in patients without denture experience (1.49) than those with (0.89). Similarly, when using the OHIP-14, the responsiveness to treatment was higher in patients without denture experience (0.83) than those with (0.60) (Tables 6 and 7).

**Table 6:** Responsiveness to treatment using GOHAI according to denture experience

Denture Experience	GOHAI	Pre-treatment	Post-treatment	Mean difference	Effect size	P
Yes	Physical function	7.55 ± 4.35	3.94 ± 4.80	3.61 ± 4.83	0.83	< 0.001^**^
	Pain and discomfort	3.00 ± 2.62	1.52 ± 2.00	1.48 ± 2.97	0.57	0.007^**^
	Psychosocial impacts	3.97 ± 4.59	1.15 ± 2.22	2.82 ± 5.20	0.61	0.004^**^
	Total score	14.52 ± 8.86	6.61 ± 6.95	7.91 ± 9.44	0.89	< 0.001^**^
No	Physical function	9.78 ± 4.63	2.81 ± 3.90	6.96 ± 5.98	1.50	< 0.001^**^
	Pain and discomfort	2.56 ± 3.29	1.15 ± 1.79	1.41 ± 3.63	0.43	0.054
	Psychosocial impacts	5.63 ± 5.19	1.04 ± 2.12	4.59 ± 5.53	0.88	< 0.001^**^
	Total score	17.96 ± 8.69	5.00 ± 5.92	12.96 ± 10.90	1.49	< 0.001^**^

**Significance at the 0.01 level (2-tailed)

**Table 7:** Responsiveness to treatment using OHIP14 according to denture experience

Denture experience	OHIP	Pre-treatment	Post-treatment	Mean difference	Effect size	P
Yes	Functional limitation	1.30 ± 1.96	0.30 ± 0.85	1.00 ± 1.90	0.51	0.005^**^
	Physical disability	1.73 ± 2.21	0.70 ± 1.19	1.03 ± 2.34	0.47	0.016^*^
	Physical pain	2.03 ± 2.69	0.55 ± 1.18	1.48 ± 2.51	0.55	0.002^**^
	Psychological discomfort	0.88 ± 1.69	0.21 ± 0.86	0.67 ± 1.73	0.39	0.034^*^
	Psychological disability	0.91 ± 1.70	0.21 ± 0.86	0.70 ± 1.91	0.41	0.044^*^
	Social disability	0.36 ± 1.45	0.00 ± 0.00	0.36 ± 1.45	0.25	0.160
	Handicap	0.52 ± 1.62	0.06 ± 0.35	0.45 ± 1.68	0.28	0.130
	Total score	7.73 ± 9.54	2.03 ± 2.76	5.70 ± 9.75	0.60	0.002^**^
No	Functional limitation	1.56 ± 1.95	0.37 ± 1.01	1.19 ± 2.24	0.61	0.011^*^
	Physical disability	2.19 ± 2.22	0.52 ± 0.98	1.67 ± 2.40	0.75	0.001^**^
	Physical pain	2.89 ± 3.48	0.33 ± 0.83	2.56 ± 3.79	0.73	0.002^**^
	Psychological discomfort	1.15 ± 1.70	0.67 ± 1.39	0.48 ± 2.28	0.28	0.282
	Psychological disability	2.19 ± 2.68	0.22 ± 0.70	1.96 ± 2.62	0.73	0.001^**^
	Social disability	1.22 ± 2.04	0.22 ± 0.70	1.00 ± 2.29	0.49	0.032^*^
	Handicap	1.33 ± 2.22	0.04 ± 0.19	1.30 ± 2.20	0.58	0.005^**^
	Total score	12.52 ± 12.29	2.37 ± 3.43	10.15 ± 13.49	0.83	0.001^**^

*Significance at the 0.05 level (2-tailed); **Significance at the 0.01 level (2-tailed)

All GOHAI and OHIP-14 items with a negative impact occurring “always” improved after prosthodontic treatment. For group A, most impacts in the GOHAI occurred in the physical function domain, followed by psychosocial domain, and finally the pain and discomfort domain. This was similar in group B but the difference between pre- and post-treatment scores of the pain and discomfort domain was not significant (*P* = 0.054). For the OHIP-14, in group A, most impacts occurred in the physical pain domain, followed by functional limitation domain, and the least were social disability and handicap domains which had no significant differences between pre- and post-treatment scores. However, in group B most impacts in the OHIP-14 occurred in the physical disability domain followed equally by physical pain and psychosocial disability domains, and the least was psychosocial discomfort which had no significant difference between pre- and post-treatment scores. In general, there was a substantial decline in impacts from all domains reported with both GOHAI and OHIP-14 tools.

## Discussion

Oral health and treatment needs of elders warrants a large amount of attention because by 2040 the number of people over the age of 60 is estimated to exceed 1 billion in developing countries, and according to a global World Health Organization estimation on oral health, approximately 30% of people aged 65–74 have no natural teeth [18].

Although dental implants are in rapidly increase in the field of prosthodontics, total edentulism is still commonly treated with conventional complete dentures, which gives importance to research investigating the impact of complete denture therapy on OHRQoL. This study assessed the impact of complete denture therapy on OHRQoL in Sudanese subjects divided according to denture experience using the GOHAI and OHIP-14 as evaluation tools. Only very few studies have been found in the literature comparing the OHRQoL of patients with and without denture experience. Bonnet et al. [19] compared the impact of treatment with different types of removable dentures, including 33 bi-maxillary complete dentures patients, on patients’ OHRQoL using GOHAI and MGDSI (McGill Denture Satisfaction Index). To the best of our knowledge, this is the first clinical study using both GOHAI and OHIP-14 to measure and compare the OHRQoL before and after treatment in patients with and without denture experience. Therefore, comparison with results of other studies will be limited. For this study, the OHIP-EDENT was not used because no Arabic version was available/validated. On the other hand, OHIP-14 was available/validated among Sudanese subjects by Khalifa et al. [13]. Although the Arabic version of GOHAI was available/validated, a cultural adaptation was necessary for this tool to be used among Sudanese subjects [8]. Although no validated Arabic version of OHIP-EDENT was available at the time of conducting the study, both GOHAI and OHIP-14 have been used widely for comparison among different populations [7, 15, 20]. Moreover, Locker et al. [21] compared both instruments as measures of OHRQoL among elderly patients living in large geriatric care center, and concluded that both measures were equally good in predicting psychological well-being and life satisfaction.

More than half the participants in current study either had a low level of education or none. These percentages are most likely not representative for the whole Sudanese population because the majority of the patients requested relatively low cost or free of charge treatments at these teaching facilities. This gives the indication that most individuals were from low economic levels and perhaps, their chances of being educated were limited. Regarding the social status, about three quarters of the participants were married, with the remaining being single, divorced, or widowed. This may reflect their psychological stability and level of overall life satisfaction. A large number of studies concluded that positive descriptions of an individual’s marriage and family situation were predictors of overall happiness or satisfaction with life [22]. By comparison, widowed, divorced, and separated people demonstrated lower satisfaction and greater unhappiness [23]. Approximately half of the sample in the present study had no previous dentures, which is indicative of the lack of approaching dental health facilities by this group of patients and reflects their need for dental treatment. Although the original GOHAI was initially assessed among subjects with 65+ years old, most of our samples were with 60+ years which is comparable with the Malay [24], Hindi [25], Japanese [26], Chinese [27], and Romanian [28] versions. Moreover, some other versions were developed among different ages [12, 29].

For the GOHAI, besides determining reliability and internal consistency, validity was determined by convergent and discriminant validity. Convergent validity was determined by identifying associations between GOHAI total score and global questions related to self-rating oral and general health status as well as DS total score. Neither GOHAI nor OHIP-14 had significant correlations with question pertaining to self-rating of oral health in group A. However, these correlations were significant in group B. It is somewhat difficult to explain this point but it might related to the fact that subjects in group A already had experience wearing dentures which in turn varied with different periods of time. Moreover, variances of factors such as denture retention, stability and esthetic (may have influenced subjects’ responses to different domains of both questionnaires. There was clear agreement between self-rated oral health and scores on the instruments used to measure OHRQoL for group B. Participants who rated their oral health as good reported a better OHRQoL, which strengthens the validity of the questionnaires used to assess OHRQoL [30].

Unexpectedly and although there were obvious differences between total scores of both instruments when related to the denture experience, these differences were not statistically significant. Only the social disability domain in OHIP-14 was significant with low score reported from group B subjects. These variations might also be related to the above mentioned reasons. The non-significant results of GOHAI are similar to that observed by Bonnet et al. [19] where they found no significant differences between GOHAI scores among patients with and without denture experience.

A significant association was found between sex and perceived OHRQoL using both OHIP 14 and GOHAI measures. Female participants presented with more impact on oral health than male participants. This finding is in accordance with previous studies such as Atchison [6], but is in contrast with another that found no association with sex [15].

More than half the respondents perceived their oral health and general health as good. GOHAI scores were significantly higher in participants with poor oral health perception and lower in those with good oral health perception. These findings were similar to those of the study by Hassel et al. [31] in which individuals rated their oral health as poor and those who were dissatisfied with their dental health had significantly poorer oral health-related quality of life than others. “Limit the type of food” and “trouble biting/chewing” were the most frequently reported oral problems in this study population. “Limit contact” and “medication for pain” were the least reported encountered oral problems. These findings correspond to those of the study by Hassel et al. in which “limit contact” was reported the least, whereas “pleased with appearance” and “worried/concerned” were more frequently reported [31]. In the present study, most impacts occurred in the physical function domain of the GOHAI, reflecting the functional needs of these elderly edentulous participants.

In general, participants had a considerably impaired level of OHRQoL before prosthetic treatment, when considering the minimal number of participants with no oral impacts. There was a highly significant reduction in GOHAI and OHIP-14 total scores post-treatment with complete denture compared with pre-treatment, indicating improved OHRQoL. This is in accordance with other studies indicating an overall improvement in OHRQoL after complete denture therapy [32, 33] therefore supporting our results. Participants with no previous denture experience generally showed greater improvement in their OHRQoL using both OHIP-14 and GOHAI instruments after complete denture placement compared with patients with previous denture experience. This could be attributed to the more drastic improvement in aesthetics, function, and speech of an individual who has received prosthetic treatment for the first time in comparison with someone with previous denture where improvement would be less obvious because they adapt to their new dentures more easily.

Most of the participants were satisfied with their prosthesis. This might be due to the fact that all clinical steps of denture construction were performed by university students under close supervision. Denture quality is regarded as a modulating factor in denture satisfaction [34] and to some extent this factor was controlled in our study. Only few patients were dissatisfied with one or more items of denture quality. These patients were mainly dissatisfied with the retention, stability, and comfort of their mandibular dentures. This finding is in agreement with those of other studies in which satisfaction with the upper dentures was greater than that with the lower dentures [35–37].

Dissatisfaction may be related to ridge anatomy and the amount of ridge resorption following extraction of teeth, especially in the mandible. Denture-bearing area as well as previous denture experience are regarded as important contributing factors to participant satisfaction with complete dentures [34]. Denture satisfaction may be a good predictor of OHRQoL, because a significant association was found between denture satisfaction and OHRQoL in the current study particularly in patients without denture experience. Patients who were satisfied with their new dentures reported improved OHRQoL. These results are similar to those from studies by Yoshida et al. [38] and Veyrune et al. [32] in which participants were satisfied with their dentures and quality of life.

Limitations of the study included the relatively small sample size and the fact that the population was limited to Khartoum State. Furthermore, no clinical evaluation of the serviced dentures was performed. To confirm the results of the current study, further studies with larger sample size and clinical evaluation of dentures to assess factors related to patient satisfaction with dentures more accurately are recommended. Similarly, use of another OHRQoL measures such as OHIP-EDENT or developing new measures better in detecting small changes would be of benefit.

## Conclusion

Perceived OHRQoL using both OHIP14 and GOHAI after complete denture therapy was better in patients without than with denture experience. Patients with previous denture experience adapted rapidly to new dentures and less significant changes were observed regarding their oral health. This factor should always been taken into account when studying the effect of complete denture among these individuals. GOHAI was more sensitive than OHIP-14 when both groups were compared according to the sociodemographic variables. Neither tool could detect the difference between both groups before or after treatment.

## Abbreviations

DS: Denture satisfaction
GOHAI: Geriatric oral health assessment index
OHIP: Oral health impact profile
OHRQoL: Oral Health-Related Quality of Life
QoL: Quality of Life

## References

[1] Locker D, Allen F (2007). What do measures of ‘oral health-related quality of life’ measure?. Community Dent Oral Epidemiol.

[2] Petersen PE (2003). The world Oral health report 2003: continuous improvement of oral health in the 21st century--the approach of the WHO global Oral health Programme. Community Dent Oral Epidemiol.

[3] Gerritsen AE, Allen PF, Witter DJ, Bronkhorst EM, Creugers NH (2010). Tooth loss and oral health-related quality of life: a systematic review and meta-analysis. Health Qual Life Outcomes.

[4] John MT, Reißmann DR, Schierz O, Allen F (2008). No significant retest effects in Oral health-related quality of life assessment using the Oral health impact profile. Acta Odontol Scand.

[5] Slade GD, Spencer AJ (1994). Development and evaluation of the Oral health impact profile. Community Dent Health.

[6] Atchison KA, Dolan TA (1990). Development of the geriatric oral health assessment index. J Dent Educ.

[7] El Osta N, Tubert-Jeannin S, Hennequin M, Bou Abboud Naaman N, El Osta L, Geahchan N (2012). Comparison of the OHIP-14 and GOHAI as measures of oral health among elderly in Lebanon. Health Qual Life Outcomes.

[8] Beaton DE, Bombardier C, Guillemin F, Ferraz MB (2000). Guidelines for the process of cross-cultural adaptation of self-report measures. Spine (Phila Pa 1976).

[9] Santucci D, Camilleri L, Kobayashi Y, Attard N (2014). Development of a Maltese version of oral health-associated questionnaires: OHIP-14, GOHAI, and the denture satisfaction questionnaire. Int J Prosthodont.

[10] Rezaei M, Rashedi V, Khedmati Morasae E. A Persian version of geriatric Oral health assessment index. Gerodontology. 2014. 10.1111/ger.12161 25319235

[11] de Souza RF, Terada AS, Vecchia MP, Regis RR, Zanini AP, Compagnoni MA (2012). Validation of the Brazilian versions of two inventories for measuring oral health-related quality of life of edentulous subjects. Gerodontology.

[12] Atieh MA (2008). Arabic version of the geriatric oral health assessment index. Gerodontology.

[13] Khalifa N, Allen PF, Abu-bakr NH, Abdel-Rahman ME (2013). Psychometric properties and performance of the Oral health impact profile (OHIP-14s-ar) among Sudanese adults. J Oral Sci.

[14] Locker D, Gibson B (2005). Discrepancies between self-ratings of and satisfaction with oral health in two older adult populations. Community Dent Oral Epidemiol.

[15] Rodakowska E, Mierzynska K, Baginska J, Jamiolkowski J (2014). Quality of life measured by OHIP-14 and GOHAI in elderly people from Bialystok, North-East Poland. BMC Oral Health.

[16] Shrout PE, Fleiss JL (1979). Intraclass correlations: uses in assessing rater reliability. Psychol Bull.

[17] Bland JM, Altman DG (1997). Statistics notes: Cronbach’s alpha. BMJ.

[18] Najman JM, Levine S (1981). Evaluating the impact of medical care and technologies on the quality of life: a review and critique. Soc Sci Med F.

[19] Bonnet G, Batisse C, Segyo JW, Veyrune JL, Nicolas E, Bessadet M (2016). Influence of the renewal of removable dentures on oral health related quality of life. Springerplus.

[20] Ikebe K, Hazeyama T, Enoki K, Murai S, Okada T, Kagawa R (2012). Comparison of GOHAI and OHIP-14 measures in relation to objective values of oral function in elderly Japanese. Community Dent Oral Epidemiol.

[21] Locker D, Matear D, Stephens M, Lawrence H, Payne B (2001). Comparison of the GOHAI and OHIP-14 as measures of the oral health-related quality of life of the elderly. Community Dent Oral Epidemiol.

[22] Suh EM (1999). National Differences in subjective well-being. Well-Being.

[23] Slade GD, Strauss RP, Atchison KA, Kressin NR, Locker D, Reisine ST (1998). Conference summary: assessing oral health outcomes--measuring health status and quality of life. Community Dent Health.

[24] Othman WN, Muttalib KA, Bakri R, Doss JG, Jaafar N, Salleh NC (2006). Validation of the geriatric Oral health assessment index (GOHAI) in the Malay language. J Public Health Dent.

[25] Mathur VP, Jain V, Pillai RS, Kalra S (2016). Translation and validation of Hindi version of geriatric Oral health assessment index. Gerodontology.

[26] Naito M, Suzukamo Y, Nakayama T, Hamajima N, Fukuhara S (2006). Linguistic adaptation and validation of the General Oral health assessment index (GOHAI) in an elderly Japanese population. J Public Health Dent.

[27] Wong MC, Liu JK, Lo EC (2002). Translation and validation of the Chinese version of GOHAI. J Public Health Dent.

[28] Murariu A, Hanganu C, Bobu L (2010). Evaluation of the reliability of the geriatric Oral health assessment index (GOHAI) in institutionalised elderly in Romania: a pilot study. OHDMBSC.

[29] Daradkeh S, Khader YS (2008). Translation and validation of the Arabic version of the geriatric Oral health assessment index (GOHAI). J Oral Sci.

[30] Dahl KE, Wang NJ, Skau I, Ohrn K (2011). Oral health-related quality of life and associated factors in Norwegian adults. Acta Odontol Scand.

[31] Hassel AJ, Steuker B, Rolko C, Keller L, Rammelsberg P, Nitschke I (2010). Oral health-related quality of life of elderly Germans--comparison of GOHAI and OHIP-14. Community Dent Health.

[32] Veyrune J, Tubert-Jeannin S, Dutheil C, Riordan P (2005). Impact of new prostheses on the oral health related quality of life of edentulous patients. Gerodontology.

[33] Sivakumar I, Sajjan S, Ramaraju AV, Rao B. Changes in Oral health-related quality of life in elderly edentulous patients after complete denture therapy and possible role of their initial expectation: a follow-up study. J Prosthodont. 2014. 10.1111/jopr.12238 25523793

[34] Van Waas MA (1990). Determinants of dissatisfaction with dentures: a multiple regression analysis. J Prosthet Dent.

[35] Čelebić A, Knezović-Zlatarić D, Papić M, Carek V, Baučić I, Stipetić J (2003). Factors related to patient satisfaction with complete denture therapy. J Gerontol Ser A Biol Med Sci.

[36] Geertman ME, van Waas MA, van’t Hof MA, Kalk W (1996). Denture satisfaction in a comparative study of implant-retained mandibular overdentures: a randomized clinical trial. Int J Oral Maxillofac Implants.

[37] Cune M, Van Kampen F, Van der Bilt A, Bosman F (2005). Patient satisfaction and preference with magnet, bar-clip, and ball-socket retained mandibular implant overdentures: a cross-over clinical trial. J Prosthet Dent.

[38] Yoshida M, Sato Y, Akagawa Y, Hiasa K (2001). Correlation between quality of life and denture satisfaction in elderly complete denture wearers. Int J Prosthodont.

